# Mental Health Consequences of the Coronavirus 2020 Pandemic (COVID-19) in Spain. A Longitudinal Study

**DOI:** 10.3389/fpsyt.2020.565474

**Published:** 2020-11-09

**Authors:** Clara González-Sanguino, Berta Ausín, Miguel Ángel Castellanos, Jesús Saiz, Aída López-Gómez, Carolina Ugidos, Manuel Muñoz

**Affiliations:** ^1^Personality, Evaluation and Clinical Psychology Department, School of Psychology, Complutense University of Madrid, Madrid, Spain; ^2^Psychobiology and Methodology in Behavioral Sciences Department, School of Psychology, Complutense University of Madrid, Madrid, Spain; ^3^Department of Social, Labor and Differential Psychology, School of Psychology, Complutense University of Madrid, Madrid, Spain; ^4^School of Psychology, Chair Against Stigma Grupo 5-Complutense University of Madrid, Complutense University of Madrid, Madrid, Spain; ^5^Personality, Evaluation and Clinical Psychology Department, School of Psychology, Chair Against Stigma Grupo 5-Complutense University of Madrid Director, Complutense University of Madrid, Madrid, Spain

**Keywords:** COVID-19, anxiety, depression, quarantine, postraumatic stress disorder

## Abstract

**Background:** Covid-19 remains a pandemic that most countries in the world are still dealing with. This is study aims to report the psychological impact of Covid-19 over time on the Spanish population.

**Methods:** A longitudinal study (*N* = 1041) was carried out with two measurements: after 2 and 5 weeks starting from the declaration of the state of emergency in Spain. The presence of depressive symptoms, anxiety, and posttraumatic stress disease (PTSD) was evaluated by means of screening tests. Sociodemographic data, variables about Covid-19, loneliness, spiritual well-being, social support, discrimination, and a sense of belonging were collected.

**Results:** The data showed how depressive symptomatology increased significantly over time, while anxiety and PTSD did not show statistically significant changes. Spiritual well-being and loneliness were the main predictors of psychological impact. A younger age was a significant predictor of depression and anxiety, while female gender was associated with anxiety and PTSD.

**Conclusions:** The impact of the pandemic is sustained over time, even increasing in depression, and vulnerable groups that need greater psychological health support could be identified.

## Introduction

Covid-19 has spread throughout the world and most countries have implemented severe health and social measures to deal with it. The pandemic, which began in China, has had a special incidence in Europe and North America, with Spain being one of the most affected countries. On 14 March, a state of emergency was declared with drastic stay-at-home measures. Since then, the population has had to remain in their homes and have only been able to go out in certain cases. From 30 March to 12 April, all non-essential work activity was suspended, which aggravated the already serious economic crisis. By 27 April 2020, a total of 210,773 confirmed cases of COVID-19 had been detected by Polymerase Chain Reaction (PCR) tests. The pandemic had caused 23,822 deaths and 102,548 people had recovered (1). At this point, Spain was the European country with the most infections, only behind the United States, and close behind Italy in terms of the total number of deaths.

The psychological consequences of this crisis are multiple. Studies are now being published, especially from China, and indicate the presence of anxiety, depression, post-traumatic stress disorders, or insomnia in a significant percentage of the population (2–6). In a previous study by this research team, we showed the short-term psychological impact of the pandemic on the Spanish population (7), revealing the presence of depression, anxiety, and post-traumatic stress. Although we are beginning to understand the most immediate effects of the pandemic on our psychological health, little is known about how this psychological impact evolves over time, with only one longitudinal study examining this to date. Wang et al. (8) studied the evolution of the psychological impact in the Chinese population 4 weeks after the start of the pandemic in 333 people, observing that the initial levels of stress, anxiety, and depression continued. This type of research, although requiring a great deal of effort, is very valuable, as it provides data that can explain the evolution of the impact on our mental health and the main predictive and protective variables involved, which will then enable more effective measures to be taken to combat the psychological effects of the pandemic.

The present study aims to longitudinally study the effects that the Covid-19 emergency and the stay-at-home measures have had on the psychological health of the Spanish population, together with the identification of the main predictors and protectors, from mid-March to the end of April 2020.

## Methods

### Procedure

The longitudinal study took place between 21 March and 27 April and used two measurements, one from 21 to 28 March and the other from 13 to 27 April. The evaluation was carried out using an online survey. This option was considered the most appropriate since it was impossible to conduct personal interviews, it reduced the cost per participant, and because this type of evaluation has shown good performance when assessing certain variables (9).

At the end of the first survey (80 items, 7-min duration approximately) an independent section was included informing the respondents that they could participate in a second evaluation, if they were willing. Those who agreed completed the second evaluation. In both cases, the signature of the informed consent and acceptance of the data protection laws were included. The study also received the approval of the Deontological Commission of the Faculty of Psychology of the Complutense University of Madrid (pr_2019_20_029).

### Participants

In the first evaluation, participants were recruited through snowball sampling (*N* = 3480) using social networks and the website www.contraelestigma.com to send the survey. For the second evaluation, those people who had agreed to participate in the study (*N* = 1,041) were directly contacted by email on a longitudinal basis.

The inclusion criteria were: (1) to be over 18 years old; (2) to be living in Spain during the Covid-19 health emergency; (3) to have agreed to participate in the second evaluation of the study.

### Instruments and Variables

The variables and instruments included in the assessment were the following:

Sociodemographic variables and variables related to Covid-19 were collected through questions developed *ad hoc*.

#### Psychological Impact

The possible symptomatology was measured using the following screening instruments: Patient Health Questionnaire-2 (PHQ-2) (10, 11). Generalized Anxiety Disorder Scale-2 (GAD-2) (12, 13). Civilian version of the Post-traumatic Stress Disorder Checklist-Reduced version (PCL-C-2) (14, 15). The PHQ-2 and the GAD-2 are brief self-report screening questionnaires that address the frequency of depressive symptoms and anxiety. They consist of two Likert-type questions ranging from 0 to 3. The PCL-C-2 was used to detect the presence of certain phenomena related to traumatic experience. The Likert-answers range from 0 to 4.

#### Discrimination

Day-to-Day Discrimination Index (InDI-D) (16). We used the main scale formed by nine Likert-type items with four response options (1–4) referring to the intersectional discrimination that can be produced by different conditions: gender, ethnicity, mental health diagnosis, and in this case, the presence of Covid-19.

#### Loneliness

Three-item version of the UCLA Loneliness Scale (UCLA-3) (17), Spanish version (18). Three items in Likert-type format with three response options.

#### Social Support

Multidimensional Scale of Perceived Social Support (EMAS) (19), adapted to Spanish (20). The scale has 12 Likert-type items with a scale of seven possible responses (1–7).

#### Spiritual Well-Being

Spiritual well-being understood as a personal search for meaning and purpose in life, in connection with a transcendent dimension of existence, and the experiences and feelings associated with that search and that connection (21). It was evaluated through the Spanish version of the Functional Assessment of Chronic Illness Therapy Spiritual Well-Being (FACIT-Sp12) (22). The answers were Likert-type from 0 to 4.

#### Self-Compassion

Self-Compassion measured by the Self-Compassion Scale (SCS) (23) Spanish version (13) evaluating how the subject usually acts toward himself in difficult moments in different dimensions. The items are Likert type (1–5).

#### Sense of Belonging

Sense of belonging was evaluated by means of four Likert-type items (1–4) previously used in other studies (24). These questions evaluated being a member of different groups.

### Analysis

The graphical representation of change for the psychological impact variables (PHQ-2, GAD-2, and PCL-C-2) was made using the standardized differences between the two measurements (T0 and T1). In addition, descriptive statistics, and coefficients for Time (and its *p*-value) from a linear mixed model are included in the results table.

To analyze the longitudinal data, linear mixed models (LMM) with random slopes (time nested to subjects) were calculated for each mental health variable. The estimation method was maximum likelihood (ML) and models were built with a step-up and theory driven approach, testing the significant change associated with fixed effects terms. As a goodness of fit index, Nakawaga's marginal pseudo-R2 statistic, which reports the percentage of variance explained by fixed effects, is provided. The analyses have been performed in R (v3.5.6) with the lme4 package.

## Results

### Characteristics of the Sample

The sample was composed of a majority of women (81%), and the 40–59 year old age group predominated (64%). About half of the sample had a partner and shared the same home with them (56%), had children in their care (55.71%) and a higher proportion had university or postgraduate studies (72%). Sixty-six percent of the persons had a job at the time of the interview, and considered that their economic situation was good-very good (66%). Fifteen percent of the sample had shown Covid-19 symptoms, while only 1% had been diagnosed, although 29% had a person or close relative with a positive diagnosis. Overall, about half of the people felt they were well-informed about the pandemic (54%), although 29% felt they had too much information.

### Longitudinal Changes in Psychological Impact

**Figure 1:**
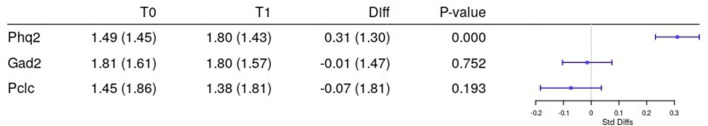
Mixed longitudinal models for depression (PHQ-2), anxiety (GAD-2), and PTSD (PCL-C-2) over time. The mixed longitudinal models presented significant time effect for the variable Phq2 (*B* = 0.31, *p* < 0.01) but not for the variables Gad2 (*B* = −0.014, *p* = 0.752) and PCL-C-2 (*B* = −0.072, *p* = 0.193). The standard deviations for the random slopes were 1.04, 1.18, and 1.49, respectively.

The results in the second assessment showed a significant increase in depression scores (*B* = 0.31, *p* < 0.01), while anxiety and PTSD scores showed no statistically significant change, only decreasing slightly (*B* = −0.014, *p* = 0.752; *B* = −0.072, *p* = 0.193). These results can be seen in Figure 1.

### Linear Mixed Models for the Psychological Impact

The regression models for the different variables showed that, for depression, the model explained 42% of the variance of the fixed effects, with the variables of spiritual well-being, loneliness, and a younger age as the main predictors. For anxiety, the model explains 30% of the fixed-effect variance, with spiritual well-being, loneliness, younger age, and female gender as the main predictors. For PTSD, the model explains 11% of the variance of the fixed effects, with spiritual well-being, loneliness, the obligation to work face-to-face, and female gender as the main predictors. This results can be seen in Table 1.

**Table 1:** Linear mixed models for depression (PHQ-2), anxiety (GAD-2), and PTSD (PCL-C-2).

	**T0**	**T1**	**PHQ2**	**GAD2**	**PCL-C-2**
Time			0.284***	−0.053	−0.084
Psychological wellness *M* (Sd)	15.61 (3.26)	15.54 (3.33)	−0.185***	−0.188***	−0.129***
Loneliness *M* (Sd)	4.43 (1.58)	4.52 (1.65)	0.221***	0.166***	0.155***
Age *M* (Sd)	39.39 (13.02)		−0.016***	−0.014***	
Gender: male *N* (%)	200 (19%)			–	–
Gender: female *N* (%)	841 (81%)			0.444***	0.673***
Not aplicable *N* (%)	413 (40%)	427 (41%)			–
Face-to-face work *N* (%)	156 (15%)	147 (14%)			0.740***
Remote working *N* (%)	474 (45%)	469 (45%)			0.061
		Time:id	1.08	1.31	1.75
		Residual	0.38	0.25	0.21
		Pseudo-R2	0.42	0.30	0.11

## Discussion

The results of the present study reflect the psychological impact of Covid-19 over time on the Spanish population. After 44 days of confinement, there was a significant increase in depressive symptoms, with no statistically significant changes in anxiety and PTSD symptoms, which even decreased slightly compared to the first evaluation (7). In China, another longitudinal study (8), showed that depression, stress, and anxiety did not present statistically significant changes, the results being consistent with our study except for depression. On the other hand, another longitudinal study carried out in Spain (25) revealed that anxiety, depression, and stress increased after 1 month. These results show discrepancies in terms of the evolution of anxiety, although it should be noted that the second evaluation of this study was made in early April, while that of the present study was made at the end, so that the greater time elapsed and the changes in the country's situation may explain these results.

Having the same or lower values for anxiety over time may be explained by the fact that the initial origin of the anxiety was based on the novelty of the situation with the consequent uncertainty and fear of contagion, a common response to a stressful situation (26). However, with the implementation of isolation measures and verification of their effectiveness, this anxiety does not intensify. Some authors have shown how the anxiety associated with Covid-19 decreases as social isolation measures such as staying home and not traveling are increased (27), which is consistent with our results. On the other hand, the increase in depression can be explained by several factors. Increased confinement time may have increased apathy and feelings of sadness, which may also be exacerbated by continued isolation and loss of social relationships and rewarding activities. In addition, it should be noted that during this second evaluation, changes at work occurred in a large part of the population. All non-essential activities were restricted, thus aggravating the economic crisis. This could also have had negative consequences on our psychological health. Some studies point to links between suicide and the economic recession of their countries, which played a more important role than fear of contagion (28, 29).

The prediction models are comparable to those found in the first evaluation of the longitudinal study (7). The main protective variable for the appearance of symptoms was spiritual well-being, while loneliness reappears as the main predictor. On the other hand, when taking into account all of the confinement time, it seems that age as a predictor variable becomes more important, as pointed out by other studies (8, 30). Young students may have initially suffered more depressive symptoms, as their lives were more affected when their daily routines were abruptly interrupted. Moreover, the initial confusion and uncertainty about the situation meant that the information received was more important in generating or reducing anxiety (5, 31). However, with the passage of time and normalization of the situation, it appears that a stronger predictor for depression and anxiety is a younger age. In addition, the female gender is a predictor of anxiety and PTSD, and this group may also be identified as more vulnerable, perhaps due to the greater burden that may arise from combining work or telework with childcare and other gender roles during the pandemic (32). The role of gender has been further studied in detail in this same sample, concluding that women have shown a greater psychological impact during confinement and highlighting the need of special attention for this group (33). On the other hand, in relation to post-traumatic symptomatology, the variables of working on site vs. teleworking or not working arise as a predictor in this model. The people who have had to work on site at their place of work during the state of emergency are those who have been on the front line of the fight against the virus, and have probably had to live through situations that can be categorized as stressful (34).

The limitations of the study include the selection of the sample using the snowball effect, which may result in an unrepresentative sample, with a higher proportion of women and younger people. Furthermore, although this is a longitudinal study, there is no control group, so the results should always be taken with caution, as other authors have pointed out (35).

The present study shows the psychological impact of Covid-19 over time on the Spanish population. The results show how, after more than 6 weeks living under an emergency situation, there has been an increase in depressive symptomatology, with anxiety and PTSD scores remaining the same. Spiritual well-being and loneliness are confirmed as the main predictors of psychological health. A younger age is associated with greater depression and anxiety, and the female gender with greater anxiety and PTSD. The results underline the importance of paying special attention to the most vulnerable groups, as well as promoting interventions to reduce loneliness and foster spiritual well-being.

## Data Availability Statement

The raw data supporting the conclusions of this article will be made available by the authors, without undue reservation.

## Ethics Statement

The studies involving human participants were reviewed and approved by Deontological Commission of the Faculty of Psychology of the Complutense University of Madrid (pr_2019_20_029). The patients/participants provided their written informed consent to participate in this study.

## Author Contributions

CG-S wrote the manuscript and participated in the design and development of the study. BA and JS reviewed the manuscript and participated in the design and development of the study. MC performed the data analysis and participated in the design and development of the study. AL-G and CU participated in the design and development of the study. MM participated in the development of the study and coordination of the group through his work as director. All authors contributed to the article and approved the submitted version.

## Conflict of Interest

The authors declare that the research was conducted in the absence of any commercial or financial relationships that could be construed as a potential conflict of interest.
